# Symptomatic Calcifications after Mastectomy: A Rare Case Report with a Review of the Literature

**DOI:** 10.3390/medicina60030399

**Published:** 2024-02-26

**Authors:** Jan Žatecký, Oldřich Coufal, Dobroslav Sekret, Matúš Peteja

**Affiliations:** 1 Department of Surgical Oncology, Masaryk Memorial Cancer Institute, 602 00 Brno, Czech Republic; coufal@mou.cz; 2 Department of Surgery, Silesian Hospital in Opava, 746 01 Opava, Czech Republicmatus.peteja@snopava.cz (M.P.); 3 The Institute of Paramedical Health Studies, Faculty of Public Policies, Silesian University, 746 01 Opava, Czech Republic; 4 Department of Surgical Oncology, Faculty of Medicine, Masaryk University, 602 00 Brno, Czech Republic

**Keywords:** calcifications, mastectomy, breast cancer, radiotherapy

## Abstract

*Introduction*: Symptomatic calcifications of the breast or skin after breast cancer surgery and adjuvant radiotherapy are a rare entity, with only a few case reports published worldwide, reducing the patient’s quality of life, whilst asymptomatic calcifications are a common finding on imaging methods. *Case presentation*: Herein, we present a rare case report of calcifications after mastectomy and post-mastectomy radiation therapy causing chronic inflammation with ulceration and fistula formation, with a two-step surgical approach consisting of excision with linear suture and excision with the reconstruction using a thoraco-epigastric flap. *Conclusions*: To our knowledge, this is the first publication proving the feasibility of this therapy in patients with symptomatic dystrophic calcifications of the skin or the breast. Moreover, the article provides an up-to-date review of published studies about symptomatic calcifications after breast cancer surgery and radiotherapy with a focus on the time of the clinical manifestation from the radiotherapy and the used radiotherapy scheme.

## 1. Introduction

Dystrophic calcifications are common incidental medical findings during X-ray examinations without any clinical difficulties in patients after breast cancer surgery and radiotherapy. On the other hand, symptomatic calcifications of the breast or skin in breast cancer patients are a rare entity with only a few case reports published worldwide [1]. The clinical manifestation is usually a painful, rigid area of the skin or breast with relapses of inflammation or abscess.

Due to the rarity of this case, the literature does not provide sufficient information about successful surgical therapy of symptomatic calcifications in breast cancer patients. Only a few case reports have been published. with different approaches and conclusions. Only one author presented surgical excision with subsequent reconstruction by a latissimus dorsi flap, but no article mentioned reconstruction by a thoraco-epigastric flap [2]. The thoraco-epigastric fascio-cutaneous flap is well-established technique used by surgeons in breast cancer patients for reconstruction after mastectomy, usually in locally advanced tumors requiring coverage of a large defect in the lower thoracic region [3,4].

Therefore, we present a unique two-step surgical approach using a thoraco-epigastric flap, along with an up-to-date literature review on calcifications after breast cancer surgery and adjuvant radiotherapy (Box 1).

Box 1 **Established Facts and Novel Insights.**

Established Facts Asymptomatic calcifications are a common finding on imaging methods after breast cancer surgery and adjuvant radiotherapy. Symptomatic calcifications of the breast or skin after breast cancer surgery and adjuvant radiotherapy are a rare entity with only a few case reports published worldwide.

 

Novel Insights A rare case report of calcifications after mastectomy and post-mastectomy radiation therapy causing chronic inflammation with ulceration and fistula formation, with a two-step surgical approach consisting of excision with linear suture and excision with the reconstruction using a thoracoepigastric flap.

To our knowledge, this is the first publication proving the feasibility of this therapy in patients with symptomatic dystrophic calcifications of the skin or the breast.

 

The article provides an up-to-date review of published studies about symptomatic calcifications after breast cancer surgery and radiotherapy.

## 2. Case Report

The patient was diagnosed with breast cancer (BC) in the left breast in 2003. Histological examination revealed lobular carcinoma and a mastectomy and axillary dissection of levels I and II was recommended by a multidisciplinary team. The patient’s comorbidities included hypertension, type 2 diabetes mellitus, hypertriglyceridemia, thrombophilia (factor V Leiden), chronic splenomegaly, euthyroid goiter and lumbo-sacral radiculopathy.

The surgery was performed on 17 March 2003, without any early post-operative complications. The final histological examination confirmed the diagnosis of lobular carcinoma with a size of 2.5 cm (pT2N1M0; triple-negative BC). Adjuvant chemotherapy was administered in the form of TEC (paclitaxel + epirubicin + cyclophosphamide) in six cycles. Adjuvant radiotherapy on the chest wall and regional lymphatics was performed from 25 August 2003 to 25 September 2003, with a total dose of 50 Gy in 25 fractions. Late complications after therapy occurred in the form of seroma in the wound and lymphedema of the left upper limb and the chest wall.

**Figure 1:**
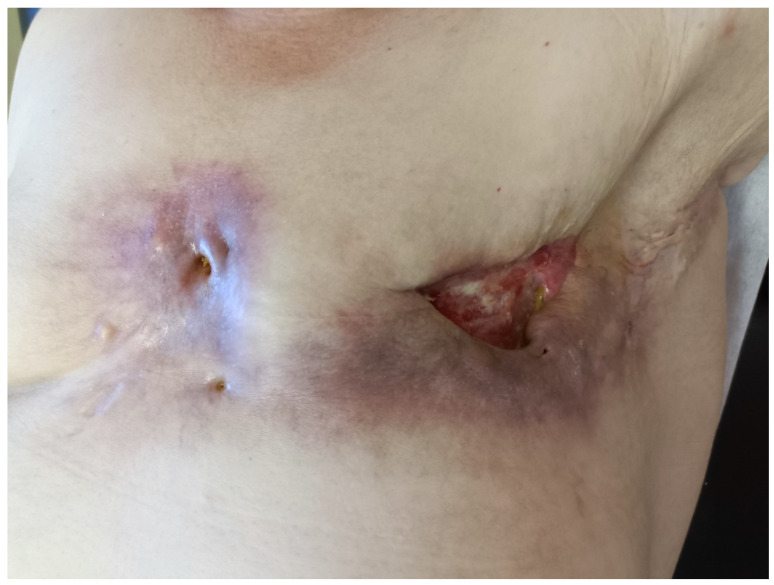
Initial local status of the patient before the surgery; multiple ulcerations and fistulae in the scar after the left mastectomy.

Despite these complications, the wound remained healed until the year 2018 (15 years after the breast cancer surgery). The first clinical manifestation of the calcifications was inflammation and pain in the wound with the evacuation of the abscess containing dystrophic calcifications. In 2018, relapses of the abscess in the wound occurred with a spontaneous discharge of the calcifications and fistulae formation. Over the next few years, the patient suffered from painful relapses and a permanent discharge of seroma, pus, and calcifications from the wound (Figure 1). The incision of the abscess was repeatedly performed, and check-ups in the surgical outpatient clinic were periodically planned. X-rays revealed multiple dystrophic calcifications in the area around the mastectomy scar with a total size of 30 × 9 cm. Over the years, the patient’s symptoms remained in the same sequence: first, the inflammation with fistula formation appeared, then the spontaneous discharge of the calcification was noted or the surgical extraction of the calcification was performed with relief of the symptoms and regression of the inflammation. When the calcification was not discharged or removed by a surgeon, the abscess appeared in the same localization. Therefore, according to repeated clinical examination, we consider the calcifications as symptomatic.

Several bacteriological examinations of the pus revealed *Streptococcus agalactiae*, *Staphylococcus aureus*, *Escherichia coli*, and *Peptostreptococcus* sp. In one of the examinations after abscess incision, *Murdochiella asaccharolytica*, *Finegoldia magna*, and *Actinomyces odontolyticus* were present.

**Figure 2:**
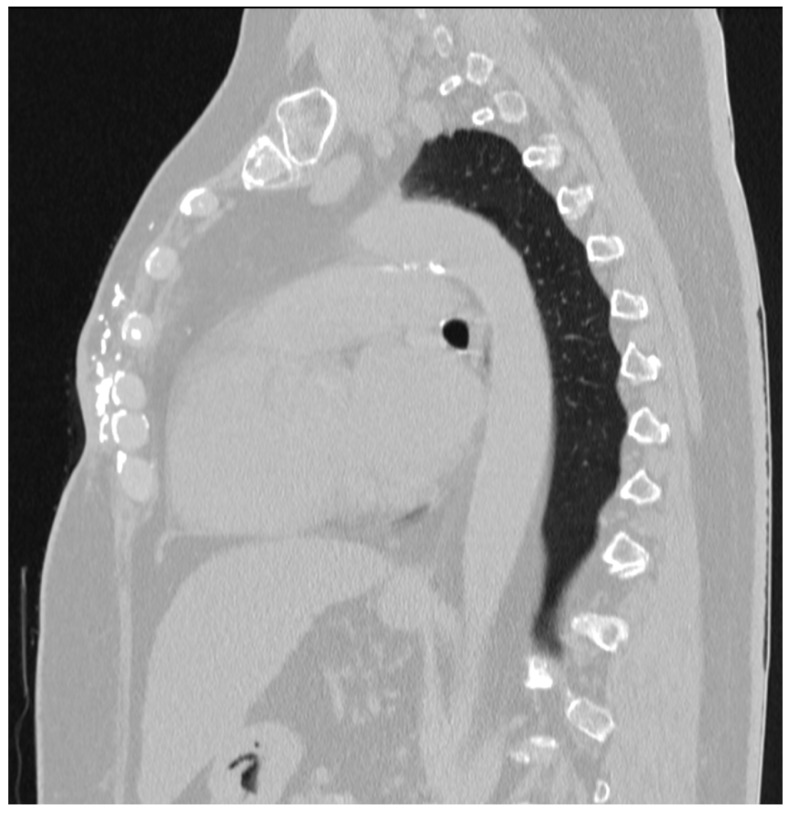
CT scan; multiple calcifications in the wound after the mastectomy ventrally to the sternum.

In 2022, the patient was referred to a breast cancer surgeon, who recommended a two-step wound excision with reconstruction. The two-step procedure was chosen because of purulent discharge from the medial part of the wound and the higher risk of postoperative inflammation of the entire wound. A CT scan was performed before the surgery to localize all calcifications and their relation to the sternum and ribs (Figure 2).

The resection was performed on 30 November 2022 and excision of the lateral part of the wound with calcifications and primary linear closure was performed. The surgery and the postoperative status were without complications, but during the check-ups, small parts of the wound healed secondarily, approximately 4 cm in total (Clavien–Dindo grade I). Histological examination of the resected skin revealed dystrophic calcifications and inflammation without any signs of cancer. Bacteriological examination of the sample taken during the surgery again revealed *Escherichia coli*.

**Figure 3:**
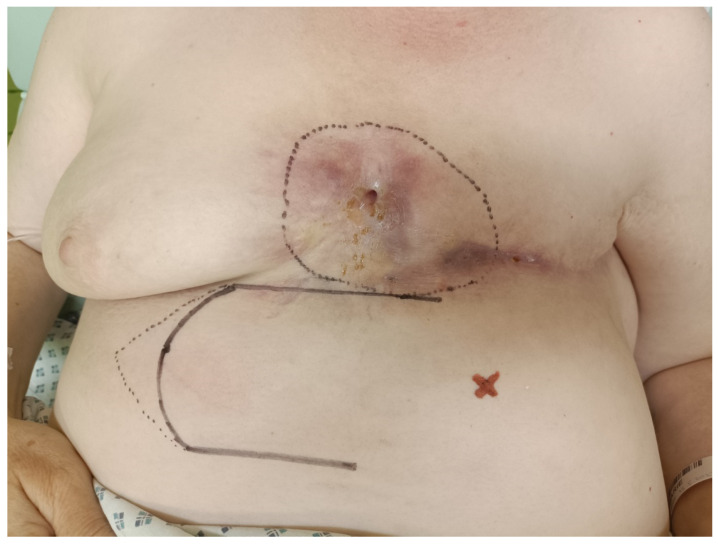
Preoperative mapping and drawing (superior epigastric artery marked by a red cross).

**Figure 4:**
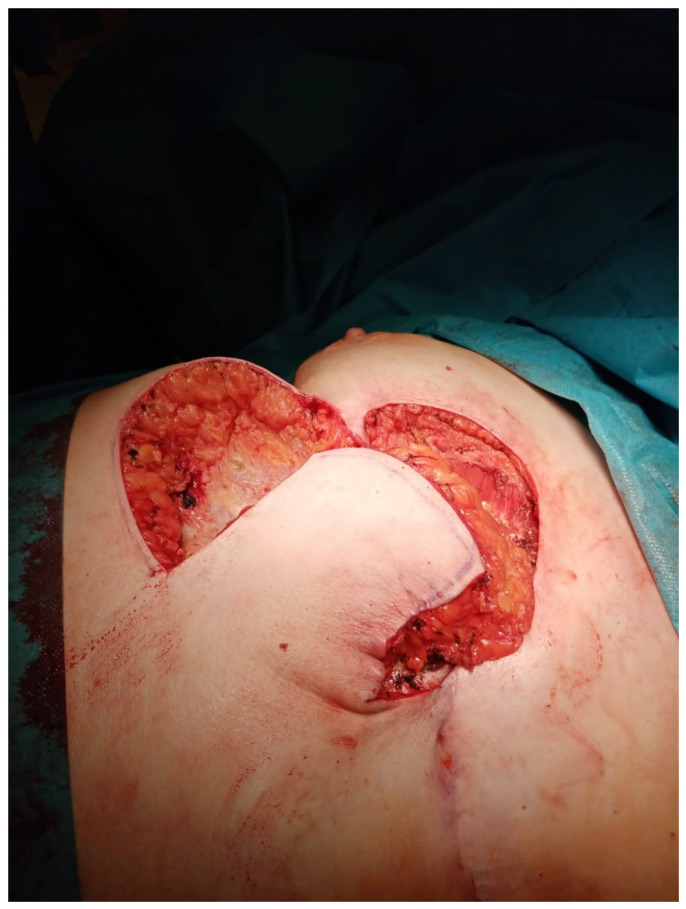
The construction of the thoraco-epigastric flap during the surgery.

The second surgery was performed on 16 May 2023, involving excision of the medial part with reconstruction using a thoraco-epigastric flap. A preoperative drawing with ultrasonography focused on the superior epigastric artery was performed (Figure 3). The patient was administered antibiotics both preoperatively and postoperatively to lower the risk of inflammation, according to the actual bacteriological examination from the fistula. During the surgery, the remaining medial part of the calcification was excised to the pectoralis major muscle and sternum. After repeated disinfection, the thoraco-epigastric flap was constructed (Figure 4). The surgery and the postoperative course were without complications and the patient was discharged from the hospital on day 6 after surgery. Histology revealed the same results as in the first step of the surgical therapy.

**Figure 5:**
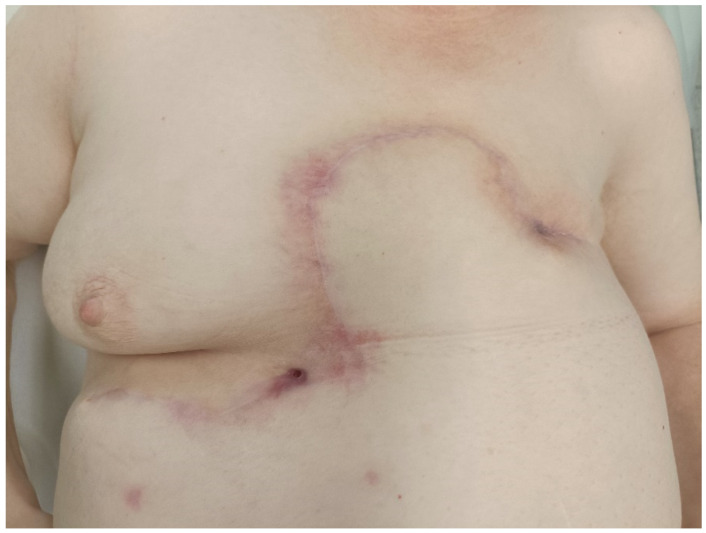
Healed scars 6 months after the surgery.

Periodical check-ups were performed, and the wound primarily healed, but repeated seroma punctures under the scar were necessary (Clavien–Dindo grade I). Three months after surgery, the patient came for a check-up due to inflammation and fever. The reason was an abscess under part of the scar that originated from the remaining seroma (Clavien–Dindo grade I). Incision and evacuation were performed, and no calcifications were found during the surgical procedure. Patient was administered antibiotics according to the bacteriological examination, which revealed *Escherichia coli* resistant to ampicillin and chloramphenicol. The patient did not suffer from any other complications (Figure 5).

## 3. Discussion

Herein, we present a rare case report of symptomatic calcifications after breast cancer surgery and adjuvant radiotherapy. Our two-step surgical approach included excision with linear suture and excision with reconstruction using a thoraco-epigastric flap. To our knowledge, this is the first publication proving the feasibility of this therapy in patients with symptomatic dystrophic calcifications of the skin or the breast.

We conducted an up-to-date review of all articles discussing symptomatic calcifications after breast cancer surgery and radiotherapy, identifying a total of eight studies encompassing 14 cases (Table 1). The articles were systematically searched in the PUBMED database using combinations of keywords, such as calcinosis, breast, calcifications, macro-calcifications, radiotherapy, and breast cancer. All references in the identified articles listed in Table 1 were double-checked for additional studies on calcifications.

The only similar case report worldwide was published by Hermann et al. The authors presented a patient with calcifications in the scar, manifesting 20 years after breast cancer surgery and radiotherapy [2]. The treatment involved antibiotics, debridement with a VAC system, and subsequent reconstruction by a latissimus dorsi flap, resulting in a successful outcome [2]. The latissimus dorsi flap is commonly chosen for immediate reconstruction after mastectomy or for defect coverage in locally advanced breast tumors [11]. Conversely, while the reconstruction of the lower thoracic region using a thoraco-epigastric flap has been documented by multiple authors, it has not been reported in patients with symptomatic calcifications [3,4]. Matrol et al. reported that the thoraco-epigastric flap yields a lower complication rate compared to Musculo-cutaneous flaps, particularly at the donor site [12]. Considering these findings and the localization of the calcifications, we opted to utilize the thoraco-epigastric flap in the presented case.

According to our review, we studied the median time from radiotherapy to clinical manifestation. Information on the time from radiotherapy to clinical manifestations of calcifications was available for 13 patients, with a median time of 16 years (ranging from 2 to 30 years). Our presented patient experienced the first clinical manifestation 15 years after adjuvant radiotherapy.

Calcifications of the breast or skin are among the late complications of surgical treatment and adjuvant radiotherapy. Other late complications include lymphedema, pulmonary complications, and second malignancies induced by radiotherapy [13]. Radiation-associated angiosarcoma is one of the most serious complications after breast radiotherapy, characterized by high local recurrence and mortality, even though it is a rare entity [14].

The historical standard for adjuvant radiotherapy after breast cancer surgery was 1.8–2.0 Gy per fraction, with a total dose of 45–60 Gy over 5–7 weeks [15]. The current standard after breast-conserving surgery recommended by NCCN guidelines is a hypo-fractionated dose of 40–42.5 Gy in 15–16 fractions to the whole breast or, in selected cases, 45–50.4 Gy in 25–28 fractions [16]. A boost to the tumor bed is recommended in patients at higher risk for recurrence with doses of 10–16 Gy in 4–8 fractions [16]. For patients after total mastectomy with a higher recurrence risk, post-mastectomy radiation therapy (PMRT), including chest wall and regional lymph node radiation, is recommended. Chest wall radiotherapy doses are 45–50.4 Gy at 1.8–2 Gy per fraction in 25–28 fractions and regional node doses are 45–50.4 Gy at 1.8–2 Gy per fraction [16]. In our review of patients with symptomatic calcifications, the doses ranged from 40–77 Gy, and the average dose with a boost was 52.9 Gy (Table 1); our presented patient received 50 Gy.

The relationship between the dose and the presence of calcifications has been studied by Herrick et al. The authors suggest that calcifications in their case report are exclusively present in regions that received more than 200 cGy/day. Therefore, they conclude that both a dose–volume effect and an effect from fractionation are important for the formation of calcification [17]. Lee et al. concluded that skin calcifications are possible late complications of radiotherapy, especially in long-term survivors after high radiation doses [9]. On the other hand, based on the data from our review, 5 out of 13 patients with clinical manifestations received a maximum dose of 40–45 Gy, not exceeding the recommended dose in current guidelines, and still developed calcifications.

The most common clinical manifestations of calcifications, as described by multiple authors, include skin edema, fibrosis, and ulcerations [1,2,5,7,9,10]. However, serious radiation sequelae, such as osteonecrosis, are rarely observed in the modern era of radiotherapy [2]. The described clinical manifestations could also be signs of breast cancer relapse; therefore, histological examination must be performed in all patients with these symptoms. In our case report, the patient experienced the same clinical symptoms, along with the formation of an abscess and a chronic fistula. On the other hand, most patients with calcifications remain asymptomatic, and Herrick et al. demonstrated that calcifications after breast cancer surgery and radiotherapy are common findings [17]. However, the literature does not provide an answer regarding what triggers clinical manifestation in such a small subgroup of patients.

The process of calcification is influenced by various factors, such as ischemia, trauma, inflammatory metabolic disorders, infections, and genetics [7]. Due to chronic inflammatory disorder and ischemia caused by radiotherapy, the dysregulation of intracellular calcium concentration in the irradiated tissue is the reason for the formation of calcium hydroxyapatite, manifesting the clinical symptoms [10].

## 4. Conclusions

Symptomatic calcifications after breast cancer surgery and adjuvant radiotherapy could be painful complications that reduce the patient’s quality of life. We presented a patient with a large area of symptomatic calcifications, successfully treated with a two-step surgical therapy. This therapy included the excision of the lateral part with primary linear closure and the excision of the medial part with reconstruction using a thoraco-epigastric flap, effectively resolving the patient’s difficulties.

## Figures and Tables

**Table 1:** Review of publications mentioning symptomatic calcifications after breast cancer surgery and adjuvant radiotherapy. (BCS = breast conserving surgery, CHT = chemotherapy, RT = radiotherapy, BC = breast cancer).

Author	Year	Type of Article	Number of Cases	Treatment of BC	RT Scheme (Gy/Fractions)	Time from RT to Clinical Manifestation (Years)	Treatment
Fosh et al. [5]	2013	case report	1	BCS, RT	50/25 + boost 10/5	13	mastectomy
Amin et al. [6]	2002	case report	1	Mastectomy, RT	45/20	N/A	N/A
Carl et al. [7]	2002	short communication	7	N/A, RT	40/-	19	N/A
				N/A, RT	77/-	11	N/A
				N/A, RT	40/-	24	N/A
				N/A, RT	40/-	7	N/A
				N/A, RT	50/-	6	N/A
				N/A, RT	50/- + boost 10/-	2	N/A
				N/A, RT	30/- + boost 15/-	16	N/A
Hermann et al. [2]	2015	case report	1	BCS, RT	48.6/- + boost 10.8/-	20	antibiotics, debridement, VAC system, latissimus dorsi flap
Perna et al. [8]	2021	correspondence	1	BCS, RT	-/-	17	surgical excision
Lee et al. [9]	2005	case report	1	Mastectomy, CHT, RT	50/20	26	patient refused recommended surgery
Lim et al. [10]	2023	case report	1	Mastectomy, RT	61/28	30	conservative
Gabani et al. [1]	2014	case report	1	Mastectomy, RT	60/30	13	wide resection with reconstruction
			14 cases		Average dose (without boost) = 49.4 Gy	Median time of clinical manifestation = 16 years	
					Average dose (with boost) = 52.9 Gy		

## Data Availability

All data generated or analysed during this study are included in this article. Further enquiries can be directed to the corresponding author.
